# Critical role of the β isoform of protein kinase C (PKCβ) in angiotensin II–induced oxidative stress in vascular smooth muscle cells

**DOI:** 10.14814/phy2.70595

**Published:** 2025-10-16

**Authors:** Hirotaka Tajima, Sayaka Naganishi, Masashi Mukohda, Mahiro Ishida, Naoki Hamada, Naoto Shigemi, Takuma Yamasaki, Sho Nakamura, Toshiyasu Matsui, Risuke Mizuno, Hiroshi Ozaki

**Affiliations:** ^1^ Laboratory of Veterinary Pharmacology, Faculty of Veterinary Medicine Okayama University of Science Imabari Ehime Japan; ^2^ Graduate School of Bioagricultural Sciences Nagoya University Nagoya Aichi Japan; ^3^ Laboratory of Veterinary Anatomy, Faculty of Veterinary Medicine Okayama University of Science Imabari Ehime Japan

**Keywords:** angiotensin II, NOX, oxidative stress, PKCβ, VSMC

## Abstract

The aim of this study was to elucidate the role of protein kinase Cβ (PKCβ) in subacute angiotensin II (Ang II)–induced oxidative stress and the mechanism of its effects on vascular smooth muscle cells (VSMCs) using a PKCβ‐knockout strategy. Both short‐term (30 min) and prolonged (24 h) treatment with Ang II increased reactive oxygen species (ROS) production through NADPH oxidase (NOX) activation, with increased phosphorylation of PKCβ at active site Ser660 in rat primary VSMCs from mesenteric artery. The increases in ROS production and NOX activity were completely abolished in VSMCs of PKCβ‐knockout rats, in which the absence of PKCβ mRNA expression was confirmed. Genomic and pharmacologic analyses indicated that prolonged treatment with Ang II increased ROS production via NF‐κB–mediated NOX1 and p22^phox^ expression via the PKCβ/ROS pathway. In vivo infusion of Ang II at a low dose (10 ng/kg/min) for 7 days increased ROS production and NOX1 and p22^phox^ expression in mesenteric artery in both male and female non‐transgenic rats, and these effects were abolished by PKCβ gene deletion. These results suggest that the PKCβ isoform is the primary regulator of oxidative stress in VSMCs in response to both acute and subacute exposure to Ang II.

## 1 INTRODUCTION

Oxidative stress plays a crucial role in the development and progression of hypertension, contributing to vascular dysfunction and blood pressure elevation. Oxidative stress is also involved in the pathogenesis of subsequent vascular proliferative diseases. Reactive oxygen species (ROS), primarily generated by NADPH oxidase (NOX), mitochondria, and uncoupled nitric oxide (NO) synthase, are major drivers of oxidative stress in hypertensive states (Harrison et al., 2021). These ROS inactivate NO, leading to reduced vasodilation, increased vascular contraction, and promotion of vascular stiffening and remodeling, which in turn promote hypertension (Laursen et al., 2001; Li et al., 2015; Park et al., 2022). ROS also contribute to sodium reabsorption in the kidneys, which increases blood volume and cardiac output (Araujo & Wilcox, 2014). In the nervous system, ROS amplify sympathetic nervous system activity, exacerbating blood pressure elevation (Lob et al., 2013). Importantly, the ensuing oxidative stress promotes inflammation and fibrosis in cardiovascular tissues, thus further aggravating hypertension (Griendling et al., 2021; Rizzoni et al., 2022). However, no approved therapies targeting ROS specifically for the treatment of hypertension in humans are currently available. Considering the essential physiologic roles of ROS, rather than broadly inhibiting all ROS, the development of drugs that specifically target excessively activated ROS‐producing enzymes or their regulatory factors may represent a more effective novel pharmacologic approach for treating chronic hypertension.

The critical role of NOX‐derived ROS production in the pathophysiology of hypertension has been clearly demonstrated in numerous studies (Dikalova et al., 2005; Matsuno et al., 2005). Among the agonists involved in cardiovascular diseases, angiotensin II (Ang II) is a key activator of NOX in hypertension (Cicalese et al., 2021); binding of Ang II to its receptor (AT1R) triggers a cascade of signaling events that primarily promote the activation of NOX1 and NOX2 (Al Ghouleh et al., 2016; Dikalov et al., 2014). This process involves the phosphorylation and activation of protein kinase C (PKC) and Rho family GTPases (e.g., Rac1), all of which are essential for the assembly of the active NOX complex at the cell membrane.

Among known PKC isoforms, PKCβ reportedly plays a particularly significant role by directly facilitating NOX1 activation (Streeter et al., 2014). As such, inhibiting PKCβ could potentially suppress hypertension and the vascular remodeling associated with chronic hypertension. However, the role of PKCβ in Ang II–induced ROS accumulation remains insufficiently investigated.

We recently demonstrated that the enhancement of vascular contraction and elevation of blood pressure induced by administration of a pressor dose of Ang II (200 ng/kg/min) for 7 days was attenuated in PKCβ knockout (KO) rats (Tajima et al., 2025). In this study, we examined the role of PKCβ in Ang II–induced oxidative stress using vascular smooth muscle cells (VSMCs) from control and PKCβ‐KO rats, with a particular focus on subacute ROS accumulation and its effects in vivo. We found that both short‐term (30 min) and prolonged (24 h) treatment with Ang II led to ROS generation, primarily via NOX activity with PKCβ activation in VSMCs from the mesenteric artery, and this effect was diminished by genetic deletion or inhibition of PKCβ. Prolonged treatment with Ang II also increased the expression of NOX1 and p22^phox^ via the PKCβ/ROS/NF‐κB pathway. Finally, ROS accumulation induced by in vivo infusion of Ang II at a low dose (10 ng/kg/min) for 7 days was completely abolished in vascular tissues of PKCβ‐KO rats.

## 2 MATERIALS AND METHODS

### 2.1 Animals

Both male and female non‐transgenic (NT) Wistar and PKCβ‐KO rats were used in this study and cared for in accordance with standards set forth by the National Institutes of Health guidelines for the care and use of experimental animals (No. 2021‐087, No. 2023‐103). The study was conducted in a clean room at a facility accredited by the Association for Assessment and Accreditation of Laboratory Animal Care (AAALAC). Animals were maintained under controlled environmental conditions (temperature: 23°C ± 1°C; humidity: 50% ± 10%) with a 12‐h light/dark cycle. Standard chow (CE‐2, CLEA Japan) and water were provided ad libitum. All procedures were approved by the Animal Care and Use Committee of Okayama University of Science.

PKCβ‐KO rats were generated using Wistar rats purchased from Japan SLC (Shizuoka, Japan), as previously described (Tajima et al., 2025). All experiments were conducted using Wistar rats obtained from the same vendor to minimize potential variability due to genetic background. In some experiments, rats (9–13 weeks old) were infused with Ang II (10 ng/kg/min) for 1 week using an osmotic minipump (Model 2001, ALZET, Cupertino, CA, USA).

### 2.2 Cell culture

VSMCs from mesenteric artery were isolated from male NT and transgenic rats (4–5 weeks old) in which the PKCβ gene was deleted. VSMCs were isolated from the first or secondary order mesenteric artery using the explant method. The cells were then suspended in high‐glucose Dulbecco's modified Eagle Medium supplemented with 10% fetal bovine serum, 1% sodium pyruvate, and 1% penicillin/streptomycin (100 units/mL) and plated in 6‐well plates at 37°C in a 5% CO_2_ incubator. After 3 days, nonadherent cells were removed by washing with phosphate‐buffered saline (PBS), and the adherent cells were cultured until reaching 90% confluence, then split for experiments. VSMCs were starved overnight before stimulation, and ROS production was measured in live cells, or protein or RNA lysates were collected and stored at −80°C until further analysis.

### 2.3 Fluorometric measurement of ROS

ROS accumulation was assessed using dihydroethidium (DHE). Mesenteric VSMCs were pretreated with the indicated agents (LY333531, CGP53353, or vehicle) for 1 h at 37°C and then incubated in the absence (control) or presence of Ang II (100 nM) for 30 min or 24 h before treatment with DHE (10 μM). DHE was determined using a fluorescence plate reader (Tecan, Männedorf, Switzerland) for in vitro analyses. The concentration of 100 nM Ang II was selected based on previous reports indicating maximal functional effects at this dose (Tajima et al., 2025).

In addition, mesenteric arteries were embedded in OCT compound (4583, Sakura Finetek Japan, Tokyo, Japan) and kept at −80°C until sectioning. Frozen sections (20 μm thick) of the mesenteric arteries were cut using a cryostat and incubated for 15 min at room temperature in PBS containing 10 μM DHE. Images were acquired using confocal microscopy (Zeiss LSM710) with a 10× objective lens at excitation/emission wavelengths of 488/568 nm, and the images were analyzed using ImageJ software.

### 2.4 Chemiluminescence analysis of NOX‐derived superoxide production

To analyze NOX‐derived superoxide production, lucigenin‐induced chemiluminescence was measured using a luminescence plate reader (Tecan). Each well of a 96‐well plate was filled with 180 μL of phosphate buffer consisting of 50 mM NaH_2_PO_4_ and NaHPO_4_, 1 mM EGTA, and 150 mM sucrose, and protein lysate (12.5–20 μg) was added in the presence of 100 μM NADPH and 500 μM lucigenin at pH 7.0. After the samples were mixed thoroughly, chemiluminescence was continuously measured for 15 min. Chemiluminescence, defined as relative light units per second (RLU/s), was measured every 10 s, and the maximal RLU/s value was compared between control and Ang II–treated samples.

### 2.5 Analysis of mitochondrial ROS accumulation

Mitochondrial ROS accumulation was assessed using a commercial fluorescent dye (MitoBright ROS Deep Red; MT16, Dojindo, Kumamoto, Japan). Following the manufacturer's instructions, mesenteric VSMCs were treated with Ang II (100 nM) for 30 min or 24 h before treatment with the dye. Mitochondrial ROS accumulation was then determined using a fluorescence plate reader (Tecan) for in vitro analyses.

### 2.6 Western blotting

VSMCs and mesenteric arteries that were cleaned of perivascular fat were snap‐frozen in liquid nitrogen. The frozen samples were then homogenized in lysis buffer consisting of 50 mmol/L Tris–HCl, 0.1 mmol/L EDTA (pH 7.5), 1% (m/vol) sodium deoxycholic acid, 1% (vol/vol) Nonidet P‐40, and 0.1% (vol/vol) SDS, supplemented with protease and phosphatase inhibitors (25955–11, 07575–51, Nacalai Tesque, Kyoto, Japan). The homogenized tissues were then subjected to rotary shaking for 1 h at 4°C and then centrifuged (20,000 *g*) for 10 min at 4°C. The protein concentration in the resulting supernatant was determined by Lowry assay. Equal amounts of total protein (7.5–20 μg) were separated by SDS‐PAGE (8%–12%) and transferred onto polyvinylidene fluoride membranes (Millipore, Burlington, MA, USA). The membranes were blocked with 5% skim milk and incubated with primary antibodies at 4°C overnight; bands were visualized by incubation with horseradish peroxidase–conjugated secondary antibodies: anti‐rabbit IgG (NA934, GE Healthcare, Chicago, IL, USA) and anti‐mouse IgG (NA931, GE Healthcare) (1:10,000 dilution, 1 h). Antibodies against PKCβ (GTX113252, GeneTex, Irvine, CA) (1:500 dilution), phospho‐PKCβ S660 (GTX59569, GeneTex) (1:300 dilution), p65 (#8242, Cell Signaling Technology, Danvers, MA) (1:500 dilution), phospho‐p65 (#3033, Cell Signaling Technology) (1:500 dilution), Akt (10176‐2‐AP, Proteintech, Rosemont, IL) (1:500 dilution), phospho‐Akt (#4060, Cell Signaling Technology) (1:300 dilution), ERK (11257‐1‐AP, Proteintech) (1:500 dilution), and phospho‐ERK (#9101, Cell Signaling Technology) (1:300 dilution) were used for these experiments. Glyceraldehyde phosphate dehydrogenase (GAPDH) was used as a loading control; the corresponding antibody (sc‐32,233) (1:5000 dilution) was obtained from Santa Cruz Biotechnology (Dallas, TX, USA).

### 2.7 Quantitative real‐time RT‐PCR (qPCR)

RNA was extracted from mesenteric VSMCs, and qPCR was performed as previously described (Mukohda et al., 2023). Briefly, using a commercial kit (Rever‐Tra Ace qPCR RT Master Mix; FSQ‐301, Toyobo, Osaka, Japan), cDNA was synthesized from 100–200 ng of total RNA extracted from the tissues using RNA Mini Kit with spin columns (74104, QIAGEN, Hilden, Germany). Each reaction was performed in duplicate. cDNA (2–5 ng) was subjected to gene expression assays using Fast SYBR Green Master Mix (A66732, Thermo Fisher Scientific, Waltham, MA, USA) along with target‐gene primers in a total volume of 10 μL. The following primers were used: rPKCβ, 5′‐GCTGGCTTCTCGTATACTAACC‐3′ (fwd) and 5′‐CAGTGGGCAATGCACTTATTC‐3′ (rev); rPKCα, 5′‐CCTGAGTATCCTGGGCTATTTG‐3′ (fwd) and 5′‐CCCTTCTTGCTCTGACTCTTAC‐3′ (rev); rPKCδ, 5′‐CCCTGAACATCTACCCTTCTTG‐3′ (fwd) and 5′‐ACTCGTGGTTCTTGATGTAGTG‐3′ (rev); rPKCε, 5′‐GCCTCTTCTTCGTCATGGAATA‐3′ (fwd) and 5′‐CAGCAGTACCCAGTCAATCTC‐3′ (rev); rGAPDH, 5′‐GGGTGTGAACCACGAGAAATA‐3′ (fwd) and 5′‐GGATGGAATTGTGAGGGAGATG‐3′ (rev); rNOX1, 5′‐CTCTGTTCTCTCCAGCCTATTC‐3′ (fwd) and 5′‐CTCCTGCAACTCCTTTCATACT‐3′ (rev); rp22^phox^, 5′‐AGTGGTACTTTGGTGCTTACTC‐3′ (fwd) and 5′‐ACCTCATCTGTCACTGGAATTG‐3′ (rev); and rNOXA1, 5′‐GCTGAGGGAACAGCTACAAA‐3′ (fwd) and 5′‐TGGCACATTCCTCCATACAC‐3′ (rev). ΔΔCT values were calculated using GAPDH as a reference gene to determine relative mRNA expression levels.

### 2.8 Blood pressure measurement using the tail‐cuff method

Systolic blood pressure was measured using the tail‐cuff method (Muromachi, Tokyo, Japan) as previously described (Seki et al., 2024). Rats were trained to reduce stress before starting measurements, and blood pressure was measured at room temperature without a heater.

### 2.9 Chemicals

Ang II (A9525), apocynin (A10809), and DHE (37,291, D7008) were purchased from Sigma‐Aldrich (St. Louis, MO, USA). LY333531 (ab219866) was purchased from Abcam (Cambridge, UK); lucigenin (CDX‐D0068‐G002) was purchased from Funakoshi (Tokyo, Japan); CGP53353 (2442) was purchased from Tocris Bioscience (Bristol, UK); Mitotempol (18796) and NADPH (9000743) were purchased from Cayman Chemical (Ann Arbor, MI, USA); and SC75741 (S7273) was purchased from Selleck Chemicals (Houston, TX, USA).

### 2.10 Statistical analyses

The number of samples was comparable between control and treatment groups in both the cell‐based and animal experiments. Results are expressed as mean ± SEM. Data were evaluated statistically using Prism software (GraphPad, San Diego, CA, USA). Where appropriate, data were compared between two groups using a two‐tailed paired or unpaired Student's *t*‐test. In other experiments, data between more than two groups were compared using two‐tailed one‐way analysis of variance with post hoc Tukey's test. Differences were considered statistically significant at *p* < 0.05.

## 3 RESULTS

### 3.1 Effect of Ang II on ROS production and phosphorylation of PKCβ

First, we investigated the extent of ROS production and the underlying mechanisms of the effects induced by the treatment of rat VSMCs with Ang II. Mesenteric VSMCs from NT rats were cultured, and we confirmed that >90% of the cells were positive for α‐smooth muscle actin (Figure S1; Figures S1–S3 are available at https://figshare.com/s/8d284fd45eae41c6a6c8).

**FIGURE 1:**
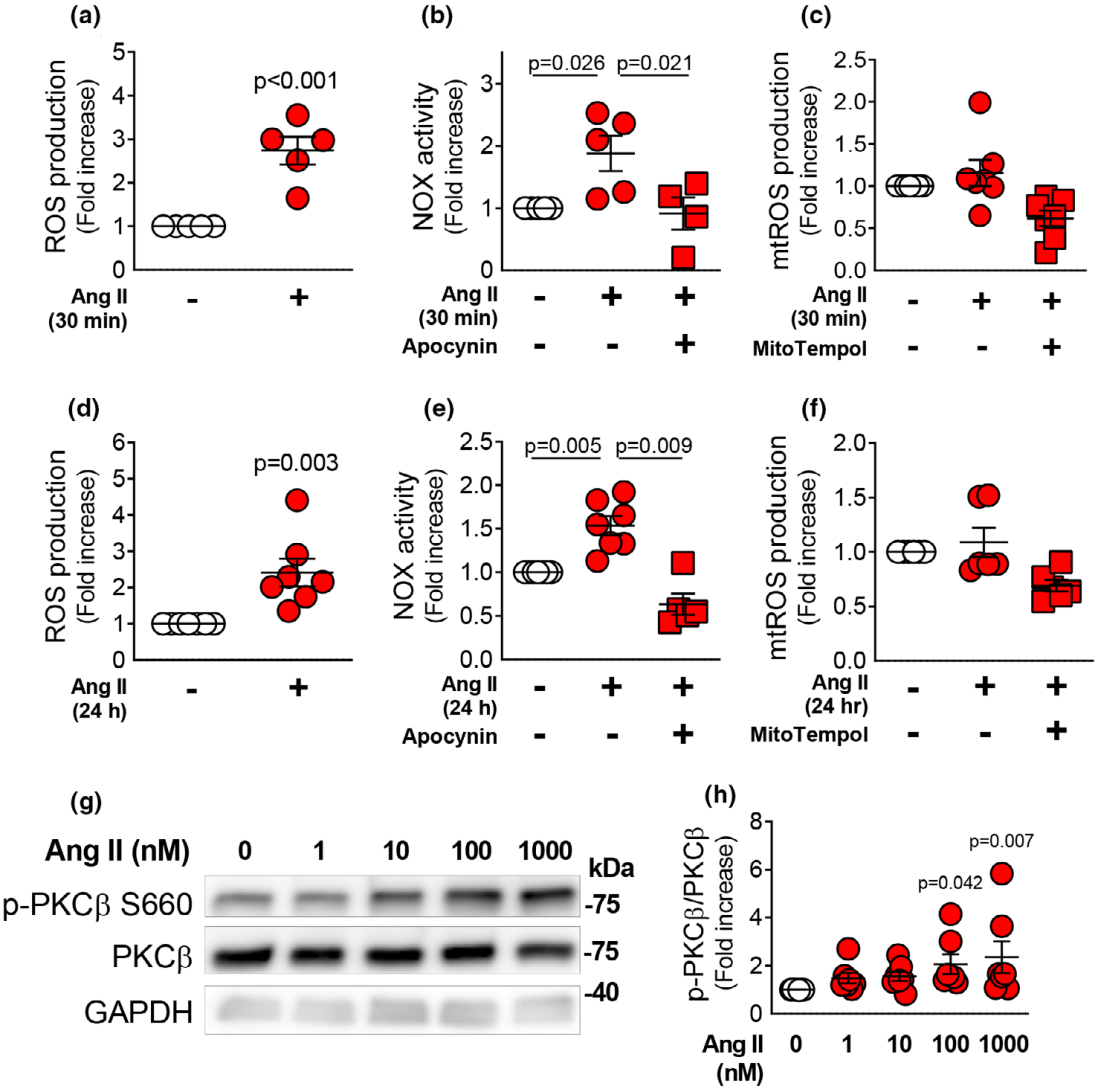
Effect of angiotensin II (Ang II) on reactive oxygen species (ROS) production and phosphorylation of protein kinase C (PKC)β in rat mesenteric vascular smooth muscle cells (VSMCs). Dihydroethidium (DHE) staining was used to detect ROS production in rat mesenteric artery primary VSMCs treated with Ang II (100 nM) for 30 min (a, *n* = 5) or 24 h (d, *n* = 7). Lucigenin assay was used to detect NADPH oxidase (NOX)‐derived superoxide production in VSMCs exposed to Ang II (100 nM) for 30 min (b, *n* = 4–5) or 24 h (e, *n* = 5–7) following pretreatment with apocynin (100 μM). Mitochondrial‐targeted fluorescent O_2_^−^ indicator was used to detect mitochondrial superoxide production in VSMCs exposed to Ang II (100 nM) for 30 min (c, *n* = 8) or 24 h (f, *n* = 8) following pretreatment with mitotempol (100 μM) as a negative control. Representative (g) and quantitative (h) Western blotting analysis of phosphorylation of PKCβ (S660), PKCβ, and GAPDH in VSMCs exposed to Ang II (0, 1, 10, 100, or 1000 nM) for 15 min (*n* = 7). Values are mean ± SEM. Exact *p*‐values are shown on the graphs.

To determine the effect of Ang II on ROS production in rat mesenteric VSMCs, live cells were stained with DHE. Short‐term treatment with Ang II (100 nM) for 30 min significantly increased ROS production in VSMCs (Figure 1a). A luciferase assay showed that treatment of VSMCs with Ang II (100 nM) for 30 min increased NOX activity, which was blocked by treatment with the NOX inhibitor, apocynin (Figure 1b). By contrast, treatment of VSMCs with Ang II (100 nM) for 30 min did not stimulate mitochondrial ROS production, as assessed using the mitochondrial‐targeted fluorescent O_2_^−^ indicator (Figure 1c). In addition, prolonged treatment with Ang II (100 nM, 24 h) also increased both ROS production (Figure 1d) and NOX activity (Figure 1e) in VSMCs, but mitochondrial ROS production was not affected (Figure 1f). These results indicate that Ang II induces ROS production by increasing non‐mitochondrial NOX activity.

We then examined whether Ang II activates PKCβ in rat mesenteric VSMCs. Treatment of VSMCs with Ang II (1–1000 nM, 15 min) resulted in phosphorylation of PKCβ at S660 in a dose‐dependent manner (Figure 1g,h), indicating that Ang II induces PKCβ activation.

### 3.2 Effect of PKCβ deletion on Ang II–induced ROS production

**FIGURE 2:**
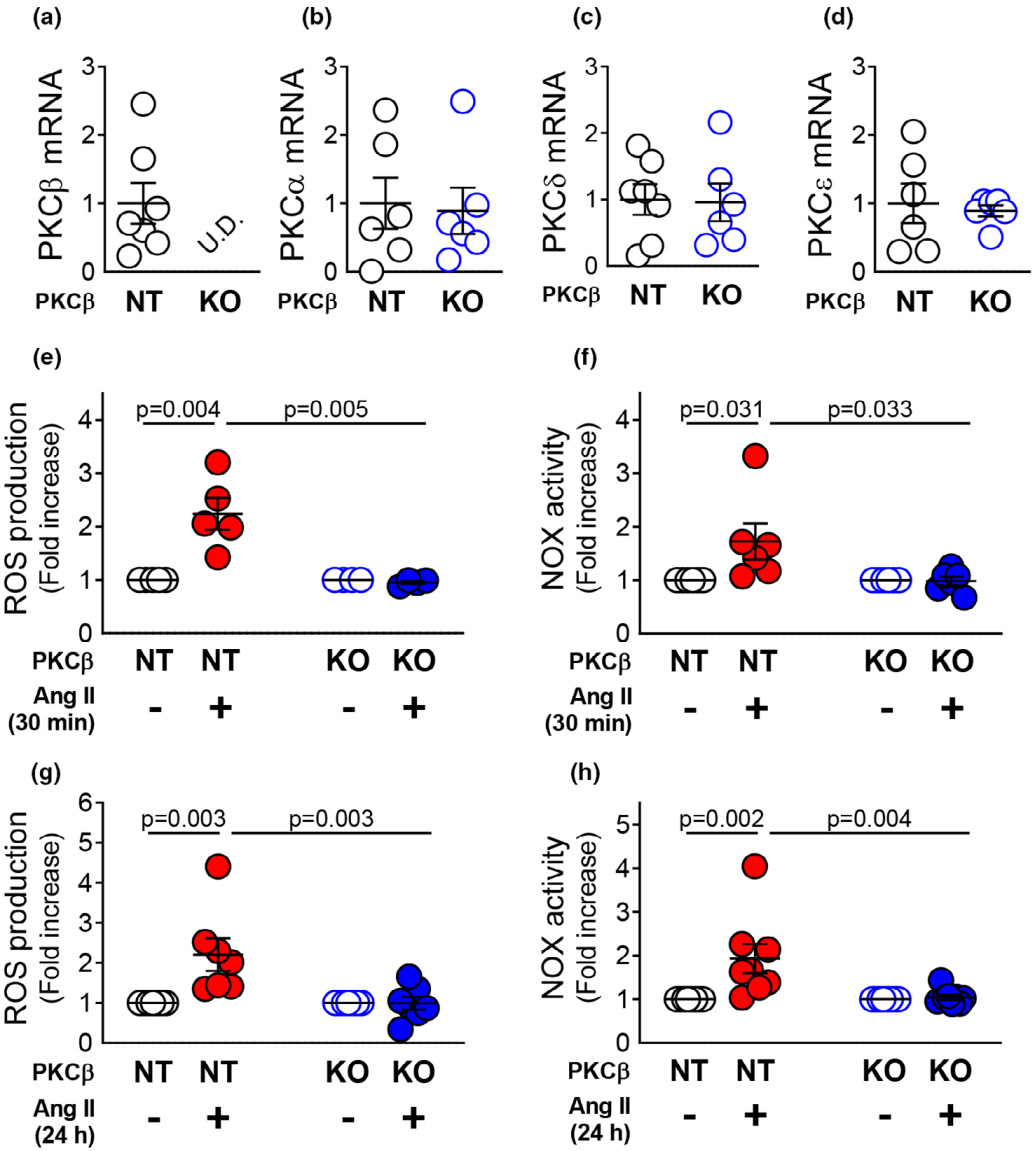
Effect of PKCβ deletion on ROS production in rat mesenteric VSMCs. Expression of PKC isoforms in primary mesenteric VSMCs from PKCβ‐knockout (KO) rats (a–d). Quantitative PCR analysis of PKCβ (a), PKCα (b), PKCδ (c), and PKCε (d) expression in primary mesenteric VSMCs of non‐transgenic (NT) and PKCβ‐KO rats (*n* = 6–7). DHE staining was used to detect ROS production in NT‐ or PKCβ‐KO–VSMCs treated with Ang II (100 nM) for 30 min (e, *n* = 5) or 24 h (g, *n* = 7). Lucigenin assay was used to detect NOX‐derived superoxide production in NT‐ or PKCβ‐KO–VSMCs treated with Ang II (100 nM) for 30 min (f, *n* = 6) or 24 h (h, *n* = 8). Values are mean ± SEM. Exact *p*‐values are shown on the graphs. U.D., undetectable.

To determine whether PKCβ is involved in Ang II–induced ROS production, we prepared mesenteric VSMCs from PKCβ‐KO rats and confirmed that expression of PKCβ mRNA could not be detected (Figure 2a). Analyses of PKCα, δ, and ε, which are highly expressed in VSMCs in addition to PKCβ (Grange et al., 1998), revealed no significant differences in the expression levels of these isoforms between NT and KO VSMCs (Figure 2b–d).

As shown in Figure 2e, short‐term treatment with Ang II (100 nM, 30 min) did not lead to increased ROS production in VSMCs from PKCβ‐KO rats. In addition, no increase in ROS production was observed following prolonged treatment with Ang II (100 nM, 24 h) in VSMCs from PKCβ‐KO rats (Figure 2g). Similarly, Ang II–induced NOX activity was greatly diminished in the cells from PKCβ‐KO rats (Figure 2f,h).

### 3.3 Effect of PKCβ inhibitor treatment on Ang II–induced ROS production

**FIGURE 3:**
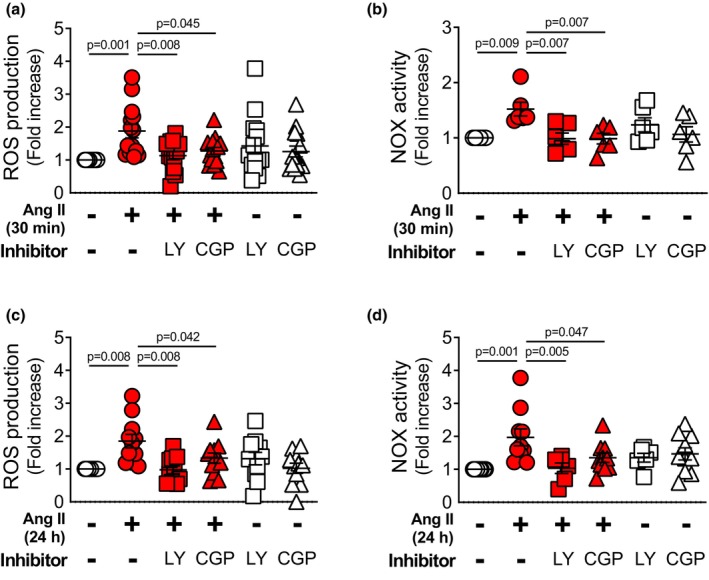
Effect of PKCβ inhibitor treatment on ROS production in rat mesenteric VSMCs. DHE staining was used to detect ROS production in VSMCs treated with Ang II (100 nM) for 30 min (a) (*n* = 14) or 24 h (c) (*n* = 11) following pretreatment with the PKCβ inhibitors LY333531 (100 nM, 1 h) or CGP53353 (100 nM, 1 h), or treatment with these PKCβ inhibitors alone. Lucigenin assay was used to detect NOX‐derived superoxide production in VSMCs exposed to Ang II (100 nM) for 30 min (b) (*n* = 6) or 24 h (d) (*n* = 6–10) following pretreatment with the PKCβ inhibitors LY333531 (100 nM, 1 h) or CGP53353 (100 nM, 1 h), or treatment with these PKCβ inhibitors alone. Values are mean ± SEM. Exact *p*‐values are shown on the graphs.

We also examined the effect of PKCβ inhibitors on Ang II–induced ROS production. In NT‐VSMCs, Ang II (100 nM, 30 min)–induced ROS production was significantly decreased by pretreatment with the PKCβ inhibitor LY333531 (10 nM, 60 min) or the relatively selective PKCβ_2_ inhibitor CGP53353 (100 nM, 60 min) (Figure 3a). Ang II (100 nM, 30 min)–induced NOX activity was also significantly decreased by pretreatment with LY333531 or CGP53353 (Figure 3b). Furthermore, pretreatment with LY333531 or CGP53353 also inhibited the increase in ROS production (Figure 3c) and NOX activity (Figure 3d) induced by prolonged Ang II treatment (100 nM, 24 h). Treatment with LY333531 or CGP53353 alone did not affect either ROS production or NOX activity compared with control cells.

### 3.4 Role of the NOX1/p22^phox^ complex in PKCβ‐mediated ROS production

**FIGURE 4:**
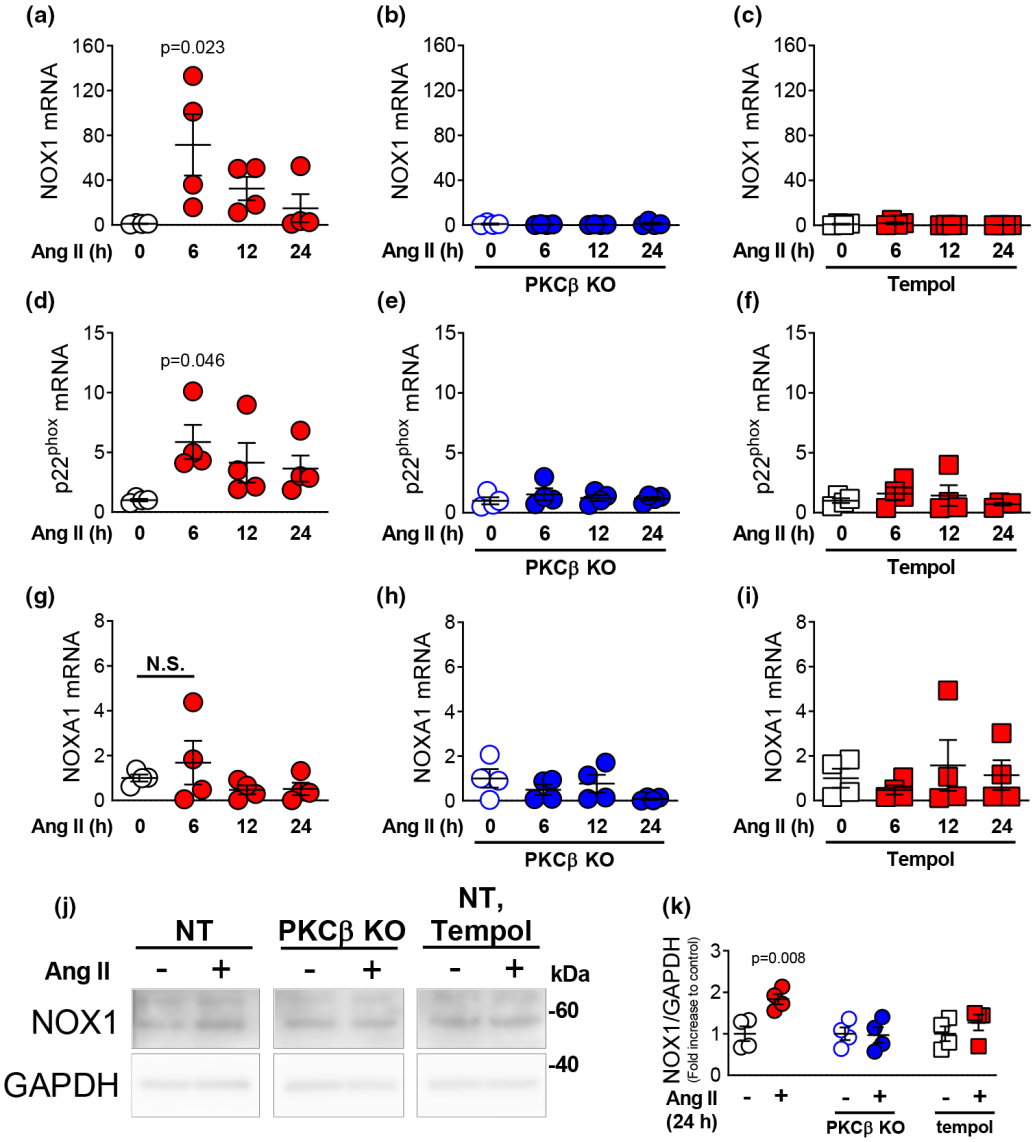
Effect of PKCβ inhibition on expression of NOX1‐related genes in rat mesenteric VSMCs. Expression of NOX1, p22^phox^, and NOX activator (NOXA)1 in primary mesenteric VSMCs from NT or PKCβ‐KO rats. Quantitative PCR analysis of NOX1 expression in NT‐VSMCs (a, c) or PKCβ‐KO–VSMCs (b) treated with Ang II (100 nM) for 6, 12, or 24 h without or with tempol (c) (*n* = 4). Quantitative PCR analysis of p22^phox^ expression in NT‐VSMCs (d, f) or PKCβ‐KO–VSMCs (e) treated with Ang II (100 nM) for 6, 12, or 24 h without or with tempol (f) (*n* = 4). Quantitative PCR analysis of NOXA1 expression in NT‐VSMCs (g, i) or PKCβ‐KO–VSMCs (h) treated with Ang II (100 nM) for 6, 12, or 24 h without or with tempol (i) (*n* = 4). Representative (j) and quantitative (k) Western blotting analysis of the expression of NOX1 in NT‐VSMCs or PKCβ‐KO–VSMCs treated with Ang II (100 nM) for 24 h without or with tempol (*n* = 4). Values are mean ± SEM. Exact *p*‐values are shown on the graphs.

The role of PKCβ in the induction of NOX activity by prolonged treatment with Ang II was examined in greater detail. Given that NOX1 and NOX4 are abundantly expressed in VSMCs (Lassegue et al., 2001; Stevenson et al., 2023), this study examined changes in the expression levels of NOX1 and associated factors. Treatment with Ang II (100 nM) for 6 h induced NOX1 mRNA expression in NT‐VSMCs (Figure 4a). By contrast, in VSMCs prepared from PKCβ‐KO rats, the Ang II–induced increase in NOX1 expression was almost completely suppressed (Figure 4b). In addition, treatment with tempol, a stable nitroxide radical exhibiting significant antioxidant and redox‐modulating properties, also attenuated Ang II–induced NOX1 expression (Figure 4c), suggesting that Ang II induces NOX1 expression through PKCβ‐derived ROS production.

We next examined the expression of p22^phox^, an essential subunit of the NOX complex and membrane‐bound protein that regulates the activity of NOX1 (Prior et al., 2016). Treatment with Ang II (100 nM) for 6–12 h significantly increased the expression of p22^phox^ mRNA (Figure 4d), but this increase was not observed in PKCβ‐KO VSMCs (Figure 4e) or NT‐derived VSMCs treated with tempol (Figure 4f). We also examined the expression of NOX activator 1 (NOXA1), which is a regulatory factor involved in the activation of NOX1 (Maehara et al., 2010). Ang II treatment did not increase NOXA1 expression levels in VSMCs from either NT or PKCβ rats (Figure 4g–i). Treatment of NT‐derived VSMCs with Ang II also increased NOX1 protein expression (Figure 4j,k); however, this increase was not observed in PKCβ‐KO VSMCs or in NT‐VSMCs treated with tempol.

### 3.5 Role of NF‐κB in PKCβ‐mediated ROS production

**FIGURE 5:**
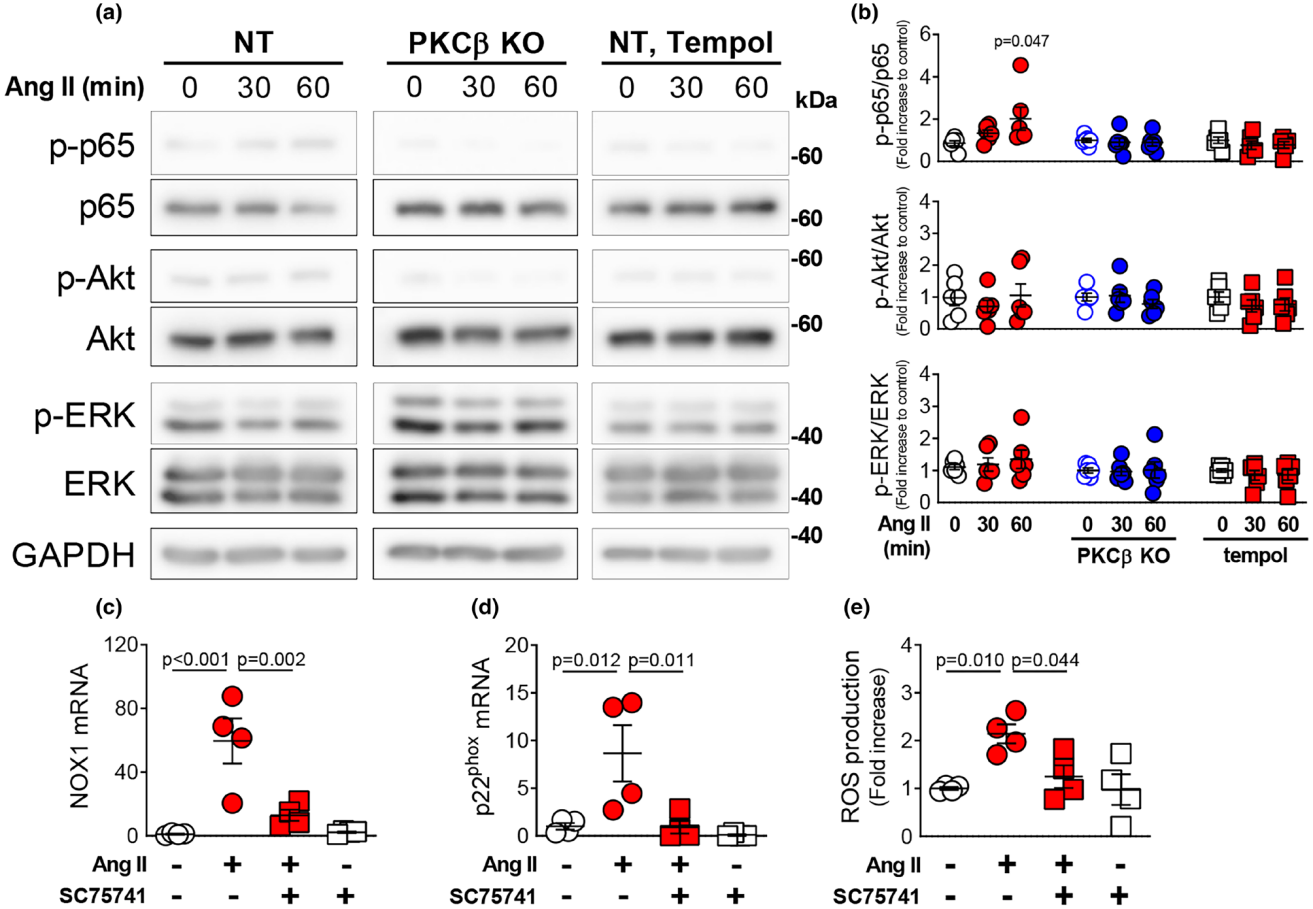
Effect of PKCβ inhibition on phosphorylation of p65, Akt, and ERK in rat mesenteric VSMCs. Representative (a) and quantitative (b) Western blotting analysis of the phosphorylation of p65 (p‐p65), Akt (p‐Akt), and ERK (p‐ERK) and expression of p65, Akt, and ERK in NT‐VSMCs or PKCβ‐KO–VSMCs treated with Ang II (100 nM) for 30 or 60 min without or with tempol (*n* = 4). Quantitative PCR analysis of NOX1 (c) and p22^phox^ (d) expression in NT‐VSMCs treated with Ang II (100 nM, 6 h) without or with the NF‐κB inhibitor SC75741 (10 μM) (*n* = 4). DHE staining to detect ROS production in NT‐VSMCs treated with Ang II (100 nM, 6 h) without or with the NF‐κB inhibitor SC75741 (10 μM) (e) (*n* = 4). Values are mean ± SEM. Exact *p*‐values are shown on the graphs.

To elucidate the mechanism underlying the Ang II–induced upregulation of NOX1 and p22^phox^ expression, we identified the associated intracellular signaling pathways. Treatment of NT‐VSMCs with Ang II (100 nM) for 60 min significantly increased the phosphorylation of p65, a subunit of NF‐κB (Figure 5a,b). By contrast, phosphorylation of p65 was blocked by PKCβ deletion or tempol treatment. In rat mesenteric artery VSMCs, Ang II treatment (100 nM, 0–60 min) did not increase the phosphorylation of ERK or Akt. Similarly, no changes in ERK or Akt phosphorylation were observed in PKCβ‐KO VSMCs or NT‐VSMCs treated with tempol.

We also examined whether Ang II–induced NF‐κB activation contributes to the upregulation of NOX1 and p22^phox^ expression. Co‐treatment with the NF‐κB inhibitor SC75741 (10 μM) attenuated Ang II (100 nM, 6 h)–induced NOX1 expression in NT‐VSMCs (Figure 5c). In addition, SC75741 co‐treatment also suppressed the Ang II (100 nM, 6 h)–induced increase in p22^phox^ expression (Figure 5d). We also confirmed that co‐treatment with SC75741 (10 μM) decreased ROS production induced by prolonged treatment with Ang II (100 nM, 24 h) (Figure 5e).

### 3.6 Effect of in vivo treatment with Ang II

Finally, we investigated whether PKCβ is involved in ROS production induced by in vivo administration of Ang II. We previously observed that infusion of Ang II at a pressor concentration (200 ng/kg/min) activates vascular PKCβ and that PKCβ deficiency suppresses Ang II–induced blood pressure elevation in male rats (Tajima et al., 2025). In this study, to control for the influence of blood pressure, we used a lower concentration of Ang II. Treatment of male Wistar rats with Ang II at a lower concentration of 10 ng/kg/min for 7 days did not alter systolic blood pressure in either NT or PKCβ‐KO rats (Figure S2a).

**FIGURE 6:**
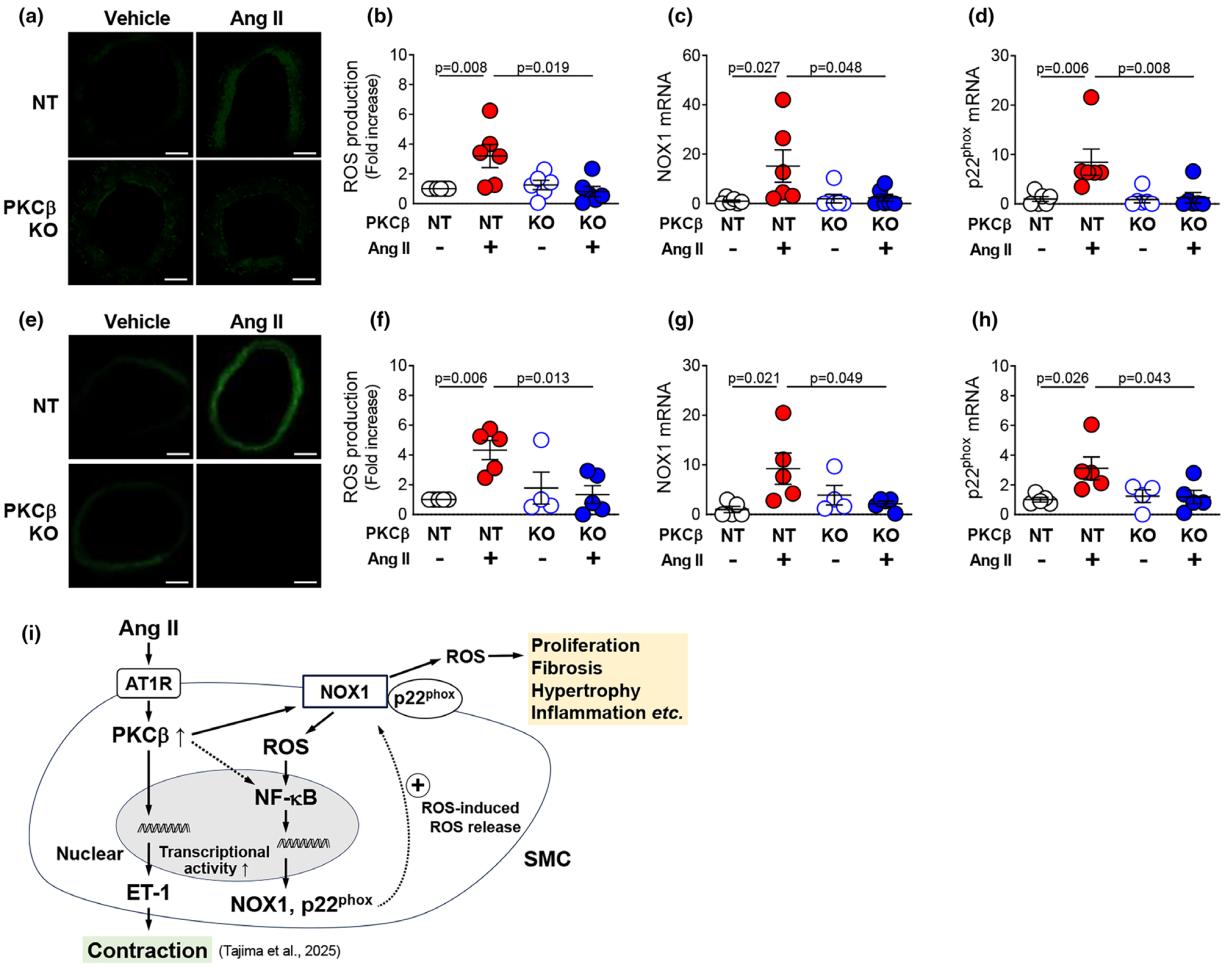
In vivo effect of PKCβ inhibition on ROS production. DHE staining was used to detect ROS production and representative (a, e) and quantified (b, f) ROS production in mesenteric artery from male (a, b) and female (e, f) NT and PKCβ‐KO rats without or with Ang II infusion (*n* = 4–6). Quantitative PCR analysis of NOX1 (c, g) and p22^phox^ (d, h) expression in mesenteric artery from male (c, d) and female (g, h) NT and PKCβ‐KO rats without or with Ang II infusion (*n* = 4–6). Schematic diagram (i). Values are mean ± SEM. Exact *p*‐values are shown on the graphs. Scale bar: 100 μm.

DHE staining revealed that ROS production increased in male NT mesenteric artery following Ang II infusion, but this effect was not observed in mesenteric artery from male PKCβ‐KO rats (Figure 6a,b). Ang II infusion increased the expression of NOX1 mRNA in mesenteric artery from male NT, but not PKCβ‐KO rats (Figure 6c). In addition, Ang II infusion increased the expression of p22^phox^ mRNA in mesenteric artery from male NT rats, but this effect was not observed in mesenteric artery from male PKCβ‐KO rats (Figure 6d). By contrast, Ang II infusion did not lead to a significant increase in NOXA1 expression in mesenteric artery from male NT or PKCβ‐KO rats (Figure S3a).

Next, we examined the effect of sex on Ang II–induced ROS accumulation. Treatment of female Wistar rats with a lower concentration of Ang II (10 ng/kg/min) for 7 days did not alter systolic blood pressure in either NT or PKCβ‐KO rats (Figure S2b). DHE staining revealed that ROS production increased in female NT mesenteric arteries following Ang II infusion, but this effect was not observed in the mesenteric artery from female PKCβ‐KO rats (Figure 6e,f). Ang II infusion increased the expression of NOX1 mRNA in the mesenteric artery from female NT, but not PKCβ‐KO rats (Figure 6g). In addition, Ang II infusion increased the expression of p22^phox^ mRNA in the mesenteric artery from female NT rats, but this effect was not observed in the mesenteric artery from female PKCβ‐KO rats (Figure 6h). By contrast, Ang II infusion did not lead to a significant increase in NOXA1 expression in the mesenteric artery from female NT or PKCβ‐KO rats (Figure S3b).

## 4 DISCUSSION

The gene encoding the serine/threonine kinase PKCβ is considered a candidate target in the treatment of diabetic vascular complications, as PKCβ plays a significant role in the inflammatory response accompanied by oxidative stress in vascular endothelial and smooth muscle cells during hyperglycemia (Durpes et al., 2015; Kong et al., 2013; Lien et al., 2021). Although oxidative stress is also recognized as a critical factor in hypertension‐induced vascular dysfunction, very few studies have investigated the role of PKCβ in hypertension, particularly in PKCβ‐deficient animals and/or under in vivo conditions. Therefore, this study examined the association between Ang II–induced oxidative stress and PKCβ in vascular cells.

### 4.1 Signaling pathway regulating Ang II–induced PKCβ activation in VSMCs

Ang II acts on VSMCs to induce vasoconstriction, proliferation and migration, fibrosis, and inflammation (Forrester et al., 2018). The Ang II–induced hypertension model is one of the most widely used models of preclinical hypertension, as it enables the establishment of a chronic hypertension state characterized by target organ damage, including vascular remodeling (Lerman et al., 2019; Okuno et al., 2023). Ang II/AT1R signaling pathways can be broadly categorized as either G‐protein dependent or G‐protein independent, with the former involving the activation of phospholipase C (PLC). The activation of multiple isoforms of PKC requires PLC activity, and Ang II is known to activate PKC via the AT1R‐G_q/11_‐PLC signaling axis (Park et al., 2022). Previous studies have reported that Ang II activates PKCβ in HEK cells as well as VSMCs derived from the aortae of mice and rats (Park et al., 2022; Shibata et al., 2014; Streeter et al., 2014). In this study, we demonstrated that Ang II treatment induces PKCβ phosphorylation in rat mesenteric artery VSMCs, thus providing further evidence that PKCβ serves as a critical mediator of Ang II–induced signaling in VSMCs.

### 4.2 Role of PKCβ in the mechanism of NOX activation and ROS amplification

In this study, we demonstrated that both short‐term and prolonged exposure of rat mesenteric VSMCs to Ang II induces NOX‐derived ROS production, which was significantly attenuated by PKCβ inhibition either through gene deletion or pharmacologic interventions. Previous studies reported that under TNF‐α stimulation, PKCβ activates NOX1 by phosphorylating Thr 429 (Streeter et al., 2014). Consistent with this observation, our findings demonstrated that activation of NOX in response to short‐term Ang II treatment (30 min) is directly mediated by PKCβ. Furthermore, prolonged exposure to Ang II (24 h) led to an increase in the expression of NOX1 and its regulatory subunit p22^phox^, which was associated with activation of the transcription factor NF‐κB. Importantly, both the activation of this signaling cascade and the upregulation of NOX1 expression were abolished by PKCβ inhibition and ROS scavenging by tempol treatment. These findings indicate that in the early phase of Ang II stimulation, PKCβ directly activates NOX, whereas in the prolonged phase, PKCβ amplifies NOX activation through the PKCβ/NOX/ROS/NF‐κB axis, promoting NOX1 and p22^phox^ transcriptional upregulation. This mechanism, often referred to as “ROS‐induced ROS release”, underscores the crucial role of PKCβ in sustaining oxidative stress in VSMCs.

By contrast, Ang II stimulation did not induce mitochondrial ROS production in this study. However, previous studies have demonstrated that Ang II induces mitochondrial ROS generation in VSMCs from aortae (Huynh et al., 2022). This discrepancy may be attributed to differences in detection methods (chemiluminescence vs. fluorescence microscopy) or cellular origin (mesenteric artery vs. aorta). The mechanism of ROS production may thus vary depending on the vascular bed examined and/or the experimental conditions.

### 4.3 In vivo analyses

In the Ang II–induced hypertension model, genetic deletion of NOX, pharmacologic inhibition, and treatment with ROS scavengers or antioxidants have been shown to attenuate endothelial dysfunction and vascular remodeling (Sedeek et al., 2009; Zhang et al., 2020). These findings suggest that ROS play a crucial role in Ang II–induced vascular damage. In this study, we demonstrated that continuous infusion of low‐dose Ang II (10 ng/kg/min for 7 days) induced ROS accumulation in vascular tissues independently of blood pressure elevation. Notably, this ROS accumulation was completely abolished in both male and female PKCβ‐deficient rats. Furthermore, Ang II infusion led to upregulation of NOX1 and p22^phox^ expression, which was also eliminated by deletion of PKCβ, indicating that Ang II–induced ROS production is highly dependent on PKCβ activity in vivo. PKCβ inhibitors such as ruboxistaurin have been tested for their effectiveness in the treatment of diabetic retinopathy and nephropathy (Kawano et al., 2021; Mochly‐Rosen et al., 2012). Further investigations into the effects of PKCβ inhibition on Ang II–induced endothelial dysfunction as well as vascular remodeling will be essential for determining the therapeutic potential of PKCβ inhibitors in the treatment of hypertension and vascular remodeling.

### 4.4 Limitations

This study has several limitations. First, VSMCs were isolated using the explant method, which may preferentially select for migratory cells and may not fully capture the in vivo heterogeneity of VSMCs (Owens et al., 2004; Tang et al., 2012). Second, although lucigenin‐based chemiluminescence assays are used to assess NOX activity, they are known to generate redox cycling artifacts that may overestimate ROS production (Janiszewski et al., 2002). Third, the concentration of Ang II used in the in vitro experiments was substantially higher than physiological levels; however, this was necessary to clearly elucidate the mechanisms of ROS production. Fourth, tail‐cuff measurements were conducted only during the light phase, which may not fully capture circadian variations in blood pressure.

### 4.5 Significance of this study

Although antioxidant therapies have been explored in several clinical trials for cardiovascular diseases, many of these trials have failed to demonstrate significant clinical benefit (Heart Outcomes Prevention Evaluation Study I et al., 2000; Singh et al., 2020; Vivekananthan et al., 2003). These findings highlight the complexity of oxidative stress mechanisms and the limitations of non‐specific antioxidant strategies in human populations. The primary source of ROS in the cardiovascular system is NOX family enzymes, which are regulated by hypertension‐ and inflammation‐associated factors such as Ang II, aldosterone, endothelin‐1, and TNF‐α (Griendling et al., 2021). In this study, we demonstrated that Ang II–induced NOX‐derived ROS accumulation was largely abolished by PKCβ inhibition in a rat model of both sexes. These findings provide further insights into the potential role of PKCβ in vascular changes associated with early stages of hypertension and suggest that PKCβ inhibition is a potentially useful therapeutic strategy for mitigating oxidative stress–induced vascular dysfunction and remodeling.

## AUTHOR CONTRIBUTIONS

H.T., S.N., M.M., and H.O. conceived and designed the research; H.T., S.N., M.M., N.S., T.Y., M.I., N.H., N.S., T.Y., S.N., and T.M. performed experiments; H.T., S.N., and M.M. analyzed data; H.T., S.N., M.M., S.N., T.M., R.M., and H.O. interpreted the experimental results; H.T., S.N., and M.M. prepared the figures and drafted the manuscript; H.O. provided extensive editing and revision of the manuscript; S.N., T.M., and R.M. edited and revised the manuscript. All authors approved the final version of the manuscript.

## FUNDING INFORMATION

This work was supported by research grants from the Mitsubishi Foundation, Takeda Science Foundation, and the Japan Society for the Promotion of Science (JSPS) Grants‐in‐Aid for Scientific Research (KAKENHI) to MM (23K23792) and RM (22K08369).

## CONFLICT OF INTEREST STATEMENT

The authors declare that no conflict of interest.

## Supporting information


**Data S1.** Supporting Information.

## Data Availability

Data supporting the conclusions of this study is available from the corresponding author upon reasonable request.

## References

[phy270595-bib-0001] Al Ghouleh, I. , Meijles, D. N. , Mutchler, S. , Zhang, Q. , Sahoo, S. , Gorelova, A. , Henrich Amaral, J. , Rodriguez, A. I. , Mamonova, T. , Song, G. J. , Bisello, A. , Friedman, P. A. , Cifuentes‐Pagano, M. E. , & Pagano, P. J. (2016). Binding of EBP50 to Nox organizing subunit p47phox is pivotal to cellular reactive species generation and altered vascular phenotype. Proceedings of the National Academy of Sciences of the United States of America, 113, E5308–E5317. 27540115 10.1073/pnas.1514161113 PMC5018796

[phy270595-bib-0002] Araujo, M. , & Wilcox, C. S. (2014). Oxidative stress in hypertension: Role of the kidney. Antioxidants & Redox Signaling, 20, 74–101. 23472618 10.1089/ars.2013.5259 PMC3880923

[phy270595-bib-0003] Cicalese, S. M. , da Silva, J. F. , Priviero, F. , Webb, R. C. , Eguchi, S. , & Tostes, R. C. (2021). Vascular stress signaling in hypertension. Circulation Research, 128, 969–992. 33793333 10.1161/CIRCRESAHA.121.318053 PMC8023761

[phy270595-bib-0004] Dikalov, S. I. , Nazarewicz, R. R. , Bikineyeva, A. , Hilenski, L. , Lassegue, B. , Griendling, K. K. , Harrison, D. G. , & Dikalova, A. E. (2014). Nox2‐induced production of mitochondrial superoxide in angiotensin II‐mediated endothelial oxidative stress and hypertension. Antioxidants & Redox Signaling, 20, 281–294. 24053613 10.1089/ars.2012.4918 PMC3887459

[phy270595-bib-0005] Dikalova, A. , Clempus, R. , Lassegue, B. , Cheng, G. , McCoy, J. , Dikalov, S. , San Martin, A. , Lyle, A. , Weber, D. S. , Weiss, D. , Taylor, W. R. , Schmidt, H. H. , Owens, G. K. , Lambeth, J. D. , & Griendling, K. K. (2005). Nox1 overexpression potentiates angiotensin II‐induced hypertension and vascular smooth muscle hypertrophy in transgenic mice. Circulation, 112, 2668–2676. 16230485 10.1161/CIRCULATIONAHA.105.538934

[phy270595-bib-0006] Durpes, M. C. , Morin, C. , Paquin‐Veillet, J. , Beland, R. , Pare, M. , Guimond, M. O. , Rekhter, M. , King, G. L. , & Geraldes, P. (2015). PKC‐beta activation inhibits IL‐18‐binding protein causing endothelial dysfunction and diabetic atherosclerosis. Cardiovascular Research, 106, 303–313. 25808972 10.1093/cvr/cvv107 PMC6279179

[phy270595-bib-0007] Forrester, S. J. , Booz, G. W. , Sigmund, C. D. , Coffman, T. M. , Kawai, T. , Rizzo, V. , Scalia, R. , & Eguchi, S. (2018). Angiotensin II signal transduction: An update on mechanisms of physiology and pathophysiology. Physiological Reviews, 98, 1627–1738. 29873596 10.1152/physrev.00038.2017 PMC6335102

[phy270595-bib-0008] Grange, J. J. , Baca‐Regen, L. M. , Nollendorfs, A. J. , Persidsky, Y. , Sudan, D. L. , & Baxter, B. T. (1998). Protein kinase C isoforms in human aortic smooth muscle cells. Journal of Vascular Surgery, 27, 919–926. 9620145 10.1016/s0741-5214(98)70273-3

[phy270595-bib-0009] Griendling, K. K. , Camargo, L. L. , Rios, F. J. , Alves‐Lopes, R. , Montezano, A. C. , & Touyz, R. M. (2021). Oxidative stress and hypertension. Circulation Research, 128, 993–1020. 33793335 10.1161/CIRCRESAHA.121.318063 PMC8293920

[phy270595-bib-0010] Harrison, D. G. , Coffman, T. M. , & Wilcox, C. S. (2021). Pathophysiology of hypertension: The mosaic theory and beyond. Circulation Research, 128, 847–863. 33793328 10.1161/CIRCRESAHA.121.318082 PMC8023760

[phy270595-bib-0011] Heart Outcomes Prevention Evaluation Study I , Yusuf, S. , Dagenais, G. , Pogue, J. , Bosch, J. , & Sleight, P. (2000). Vitamin E supplementation and cardiovascular events in high‐risk patients. The New England Journal of Medicine, 342, 154–160. 10639540 10.1056/NEJM200001203420302

[phy270595-bib-0012] Huynh, D. T. N. , Jin, Y. , Van Nguyen, D. , Myung, C. S. , & Heo, K. S. (2022). Ginsenoside Rh1 inhibits angiotensin II‐induced vascular smooth muscle cell migration and proliferation through suppression of the ROS‐mediated ERK1/2/p90RSK/KLF4 signaling pathway. Antioxidants (Basel), 11, 643. 35453328 10.3390/antiox11040643 PMC9030830

[phy270595-bib-0013] Janiszewski, M. , Souza, H. P. , Liu, X. , Pedro, M. A. , Zweier, J. L. , & Laurindo, F. R. (2002). Overestimation of NADH‐driven vascular oxidase activity due to lucigenin artifacts. Free Radical Biology & Medicine, 32, 446–453. 11864784 10.1016/s0891-5849(01)00828-0

[phy270595-bib-0014] Kawano, T. , Inokuchi, J. , Eto, M. , Murata, M. , & Kang, J. H. (2021). Activators and inhibitors of protein kinase C (PKC): Their applications in clinical trials. Pharmaceutics, 13, 1748. 34834162 10.3390/pharmaceutics13111748 PMC8621927

[phy270595-bib-0015] Kong, L. , Shen, X. , Lin, L. , Leitges, M. , Rosario, R. , Zou, Y. S. , & Yan, S. F. (2013). PKCbeta promotes vascular inflammation and acceleration of atherosclerosis in diabetic ApoE null mice. Arteriosclerosis, Thrombosis, and Vascular Biology, 33, 1779–1787. 23766264 10.1161/ATVBAHA.112.301113 PMC3865290

[phy270595-bib-0016] Lassegue, B. , Sorescu, D. , Szocs, K. , Yin, Q. , Akers, M. , Zhang, Y. , Grant, S. L. , Lambeth, J. D. , & Griendling, K. K. (2001). Novel gp91(phox) homologues in vascular smooth muscle cells: Nox1 mediates angiotensin II‐induced superoxide formation and redox‐sensitive signaling pathways. Circulation Research, 88(9), 888–894. 11348997 10.1161/hh0901.090299

[phy270595-bib-0017] Laursen, J. B. , Somers, M. , Kurz, S. , McCann, L. , Warnholtz, A. , Freeman, B. A. , Tarpey, M. , Fukai, T. , & Harrison, D. G. (2001). Endothelial regulation of vasomotion in apoE‐deficient mice: Implications for interactions between peroxynitrite and tetrahydrobiopterin. Circulation, 103, 1282–1288. 11238274 10.1161/01.cir.103.9.1282

[phy270595-bib-0018] Lerman, L. O. , Kurtz, T. W. , Touyz, R. M. , Ellison, D. H. , Chade, A. R. , Crowley, S. D. , Mattson, D. L. , Mullins, J. J. , Osborn, J. , Eirin, A. , Reckelhoff, J. F. , Iadecola, C. , & Coffman, T. M. (2019). Animal models of hypertension: A scientific Statement from the American Heart Association. Hypertension, 73, e87–e120. 30866654 10.1161/HYP.0000000000000090 PMC6740245

[phy270595-bib-0019] Li, L. , Feng, D. , Luo, Z. , Welch, W. J. , Wilcox, C. S. , & Lai, E. Y. (2015). Remodeling of afferent arterioles from mice with oxidative stress does not account for increased contractility but does limit Excessive Wall stress. Hypertension, 66, 550–556. 26101341 10.1161/HYPERTENSIONAHA.115.05631 PMC4537373

[phy270595-bib-0020] Lien, C. F. , Chen, S. J. , Tsai, M. C. , & Lin, C. S. (2021). Potential role of protein kinase C in the pathophysiology of diabetes‐associated atherosclerosis. Frontiers in Pharmacology, 12, 716332. 34276388 10.3389/fphar.2021.716332 PMC8283198

[phy270595-bib-0021] Lob, H. E. , Schultz, D. , Marvar, P. J. , Davisson, R. L. , & Harrison, D. G. (2013). Role of the NADPH oxidases in the subfornical organ in angiotensin II‐induced hypertension. Hypertension, 61, 382–387. 23248154 10.1161/HYPERTENSIONAHA.111.00546 PMC3678909

[phy270595-bib-0022] Maehara, Y. , Miyano, K. , Yuzawa, S. , Akimoto, R. , Takeya, R. , & Sumimoto, H. (2010). A conserved region between the TPR and activation domains of p67phox participates in activation of the phagocyte NADPH oxidase. The Journal of Biological Chemistry, 285, 31435–31445. 20679349 10.1074/jbc.M110.161166 PMC2951218

[phy270595-bib-0023] Matsuno, K. , Yamada, H. , Iwata, K. , Jin, D. , Katsuyama, M. , Matsuki, M. , Takai, S. , Yamanishi, K. , Miyazaki, M. , Matsubara, H. , & Yabe‐Nishimura, C. (2005). Nox1 is involved in angiotensin II‐mediated hypertension: A study in Nox1‐deficient mice. Circulation, 112, 2677–2685. 16246966 10.1161/CIRCULATIONAHA.105.573709

[phy270595-bib-0024] Mochly‐Rosen, D. , Das, K. , & Grimes, K. V. (2012). Protein kinase C, an elusive therapeutic target? Nature Reviews. Drug Discovery, 11, 937–957. 23197040 10.1038/nrd3871 PMC3760692

[phy270595-bib-0025] Mukohda, M. , Yano, T. , Matsui, T. , Nakamura, S. , Miyamae, J. , Toyama, K. , Mitsui, R. , Mizuno, R. , & Ozaki, H. (2023). Treatment with Ligilactobacillus murinus lowers blood pressure and intestinal permeability in spontaneously hypertensive rats. Scientific Reports, 13, 15197. 37709803 10.1038/s41598-023-42377-7 PMC10502128

[phy270595-bib-0026] Okuno, K. , Torimoto, K. , Kuroda, R. , Cicalese, S. M. , Okuno, Y. , Kono, R. , Marumoto, S. , Utsunomiya, H. , & Eguchi, S. (2023). Infused juice concentrate of Japanese plum Prunus mume attenuates inflammatory vascular remodeling in a mouse model of hypertension induced by angiotensin II. Hypertension Research, 46, 1923–1933. 37308550 10.1038/s41440-023-01332-9

[phy270595-bib-0027] Owens, G. K. , Kumar, M. S. , & Wamhoff, B. R. (2004). Molecular regulation of vascular smooth muscle cell differentiation in development and disease. Physiological Reviews, 84, 767–801. 15269336 10.1152/physrev.00041.2003

[phy270595-bib-0028] Park, J. M. , Do, V. Q. , Seo, Y. S. , Kim, H. J. , Nam, J. H. , Yin, M. Z. , Kim, H. J. , Kim, S. J. , Griendling, K. K. , & Lee, M. Y. (2022). NADPH oxidase 1 mediates acute blood pressure response to angiotensin II by contributing to calcium influx in vascular smooth muscle cells. Arteriosclerosis, Thrombosis, and Vascular Biology, 42, e117–e130. 35354309 10.1161/ATVBAHA.121.317239

[phy270595-bib-0029] Prior, K. K. , Leisegang, M. S. , Josipovic, I. , Lowe, O. , Shah, A. M. , Weissmann, N. , Schroder, K. , & Brandes, R. P. (2016). CRISPR/Cas9‐mediated knockout of p22phox leads to loss of Nox1 and Nox4, but not Nox5 activity. Redox Biology, 9, 287–295. 27614387 10.1016/j.redox.2016.08.013 PMC5021817

[phy270595-bib-0030] Rizzoni, D. , De Ciuceis, C. , Szczepaniak, P. , Paradis, P. , Schiffrin, E. L. , & Guzik, T. J. (2022). Immune system and microvascular remodeling in humans. Hypertension, 79, 691–705. 35098718 10.1161/HYPERTENSIONAHA.121.17955

[phy270595-bib-0031] Sedeek, M. , Hebert, R. L. , Kennedy, C. R. , Burns, K. D. , & Touyz, R. M. (2009). Molecular mechanisms of hypertension: Role of Nox family NADPH oxidases. Current Opinion in Nephrology and Hypertension, 18, 122–127. 19430333 10.1097/MNH.0b013e32832923c3

[phy270595-bib-0032] Seki, M. , Mukohda, M. , Tajima, H. , Morikita, N. , Imai, R. , Itaya, K. , Mizuno, R. , & Ozaki, H. (2024). Long‐term treatment with the streptococcal exotoxin streptolysin O inhibits vascular smooth muscle contraction by inducing iNOS expression in endothelial cells. Journal of Pharmacology and Experimental Therapeutics, 392(2), 100011. 40023601 10.1124/jpet.124.002121

[phy270595-bib-0033] Shibata, S. , Arroyo, J. P. , Castaneda‐Bueno, M. , Puthumana, J. , Zhang, J. , Uchida, S. , Stone, K. L. , Lam, T. T. , & Lifton, R. P. (2014). Angiotensin II signaling via protein kinase C phosphorylates Kelch‐like 3, preventing WNK4 degradation. Proceedings of the National Academy of Sciences of the United States of America, 111, 15556–15561. 25313067 10.1073/pnas.1418342111 PMC4217463

[phy270595-bib-0034] Singh, S. , Nautiyal, A. , & Belk, K. W. (2020). Real world outcomes associated with Idarucizumab: Population‐based retrospective cohort study. American Journal of Cardiovascular Drugs, 20, 161–168. 31332727 10.1007/s40256-019-00360-6

[phy270595-bib-0035] Stevenson, M. D. , Vendrov, A. E. , Yang, X. , Chen, Y. , Navarro, H. A. , Moss, N. , Runge, M. S. , Arendshorst, W. J. , & Madamanchi, N. R. (2023). Reactivity of renal and mesenteric resistance vessels to angiotensin II is mediated by NOXA1/NOX1 and superoxide signaling. American Journal of Physiology. Renal Physiology, 324, F335–F352. 36759130 10.1152/ajprenal.00236.2022 PMC10026993

[phy270595-bib-0036] Streeter, J. , Schickling, B. M. , Jiang, S. , Stanic, B. , Thiel, W. H. , Gakhar, L. , Houtman, J. C. , & Miller, F. J., Jr. (2014). Phosphorylation of Nox1 regulates association with NoxA1 activation domain. Circulation Research, 115, 911–918. 25228390 10.1161/CIRCRESAHA.115.304267 PMC5025877

[phy270595-bib-0037] Tajima, H. , Morikita, N. , Mukohda, M. , Nakamura, S. , Seki, M. , Imai, R. , Saito, F. , Mizuno, R. , & Ozaki, H. (2025). Enhanced vascular contraction induced by exposure to angiotensin II mediated by endothelin‐1 biosynthesis following PKCbeta activation. American Journal of Physiology Heart and Circulatory Physiology, 328(3), H484–H495. 39887322 10.1152/ajpheart.00541.2024

[phy270595-bib-0038] Tang, Z. , Wang, A. , Yuan, F. , Yan, Z. , Liu, B. , Chu, J. S. , Helms, J. A. , & Li, S. (2012). Differentiation of multipotent vascular stem cells contributes to vascular diseases. Nature Communications, 3, 875. 10.1038/ncomms1867 PMC3538044 22673902

[phy270595-bib-0039] Vivekananthan, D. P. , Penn, M. S. , Sapp, S. K. , Hsu, A. , & Topol, E. J. (2003). Use of antioxidant vitamins for the prevention of cardiovascular disease: Meta‐analysis of randomised trials. Lancet, 361, 2017–2023. 12814711 10.1016/S0140-6736(03)13637-9

[phy270595-bib-0040] Zhang, Y. , Murugesan, P. , Huang, K. , & Cai, H. (2020). NADPH oxidases and oxidase crosstalk in cardiovascular diseases: Novel therapeutic targets. Nature Reviews Cardiology, 17, 170–194. 31591535 10.1038/s41569-019-0260-8 PMC7880919

