# Supplementary material for: Critical role of the β isoform of protein kinase C (PKCβ) in angiotensin II–induced oxidative stress in vascular smooth muscle cells
Source: Physiol Rep. 2025 Oct 16;13(20):e70595. doi: 10.14814/phy2.70595 (PMC12531348; doi:10.14814/phy2.70595)
Supplement: Supplementary file 1 — Data S1. Supporting Information. [file PHY2-13-e70595-s001.doc]

# Online Supplement

# Critical role of the β isoform of protein kinase C (PKCβ) in angiotensin II–induced oxidative stress in vascular smooth muscle cells

# Hirotaka Tajima*, Sayaka Naganishi*, Masashi Mukohda, Mahiro Ishida, Naoki Hamada, Naoto Shigemi, Takuma Yamasaki, Sho Nakamura, Toshiyasu Matsui, Risuke Mizuno, Hiroshi Ozaki

# * Tajima H and Naganishi S are equal contributors to this work and designated as co-first authors.

# Contents

# 1. Supplemental Figure

**Supplemental Fig 1.** Representative immunofluorescence staining of α-smooth muscle actin (green) in primary smooth muscle cells isolated from the mesenteric arteries of Wistar rats. Nuclei were counterstained with DAPI (blue). Scale bar: 50 μm.

**Supplemental Fig 2.** Systolic blood pressure (SBP) was measured at day 7 of Ang II infusion (10 ng/kg/min) or sham control in male non-transgenic (NT) and PKCβ-KO rats (A, n=6). SBP was measured at day 7 of Ang II infusion (10 ng/kg/min) or sham control in female NT and PKCβ-KO rats (B, n=4-5). SBP was also measured at day 7 Values are shown as mean ± SEM. N.S.: not significant..

**Supplemental Fig 3.** Quantitative PCR analysis of NOXA1 expression in aorta from male NT and PKCβ-KO rats without or with Ang II infusion (A, n=6). Quantitative PCR analysis of NOXA1 expression in aorta from female NT and PKCβ-KO rats without or with Ang II infusion (B, n=4-5). Values are shown as mean ± SEM.
